# Total Water Intake and Urine Measures of Hydration in Adult Dogs Drinking Tap Water or a Nutrient-Enriched Water

**DOI:** 10.3389/fvets.2018.00317

**Published:** 2018-12-18

**Authors:** Brian M. Zanghi, Cari L. Gardner

**Affiliations:** Nestlé Purina Research, St. Louis, MO, United States

**Keywords:** canine, water supplement, hydration, drinking, water ingestion

## Abstract

Water intake and urine measures were evaluated in dogs offered tap water (TW) or a nutrient-enriched water (NW) supplement while fed dry food with *ad libitum* TW in a bucket. Baseline (day-7) urine specific gravity (U_SG_) was analyzed from healthy, adult small breed dogs (*n* = 21; 2–11 years). Dogs (*N* = 16) were selected with ≥1.015 U_SG_, then equally divided into 2 groups balanced for U_SG_. Groups received either TW or NW in a bowl for 56 days. Dose for each dog was 0.5:1 water-to-calorie ratio (mL:kcal ME/d) from days 1–49 to evaluate sustained intake of a moderate volume, or 2:1 water-to-calorie ratio from days 50–56 to evaluate short-term intake of a large volume, based on baseline food calorie intake. Daily food calorie and total liquid intake (TLI; g/d; sum of NW or TW in a bowl and bucket water) was used to calculate weekly intake. U_SG_ was measured on days −7, 14, 42, 56. Calorie intake was not different (*P* > 0.49). A significant (*P* < 0.001) time-by-treatment interaction resulted for TLI with baseline similar between groups and no difference between weeks for the TW group. Following baseline, NW group had increased (*P* < 0.05) TLI every week, except for week 2 (*P* = 0.07). A significant (*P* < 0.002) time-by-treatment interaction resulted for U_SG_, with baseline similar between groups and no difference between sampling days for the TW group (varied by ≤ 0.006 g/mL), whereas NW group was lower (*P* < 0.01) on days 42 (1.018 g/mL) and 56 (1.014 g/mL) vs. baseline (1.026 g/mL). This study indicates that all dogs offered the NW supplement increased their TLI and produced a more dilute urine, which suggests an improvement in indices associated with chronic hydration.

## Introduction

Estimates of daily water requirements have been reported for dogs (1). However, no consensus exists for how to define optimal hydration, optimal water intake volume, or the overall impact of adequate hydration on health in dogs. This results in the continued reliance on a nutritional recommendation of always having fresh water available for the pet's own desire to ingest water and establish individual eu-hydration (1). Only a basic and limited framework of published research exists on water intake, water balance, and urine variables in dogs (1, 2). Daily water intake volume has been reported as mL/kg of body weight, mL/kg of dry matter ingested, and water-to-calorie intake ratio as mL/kcal of ME ingested (1). All of these methods account for the intake of water from a combination of sources including food moisture, free water consumption (drinking), and metabolic water. In general, the daily water-to-calorie intake ratio for a healthy dog's water need has been conservatively estimated to be 1.0:1.0 mL water:kcal of ME (1), and this metric seems ideally suited as a means of estimating the daily water need because calorie intake can dramatically change over time (weeks to months) with varying levels of physical activity associated with work or exercise, yet BW can remain the same. However, some exceptions have been reported for dogs living and performing ultraendurance exercise in extremely cold climates (3).

Although group mean daily water intake has been reported in healthy dogs, it is not clear how an individual dog's water intake influences their corresponding urine concentration measured by specific gravity (U_SG_) or osmolality (U_osm_), as well as various urine analyte concentrations. Unlike cats, but similar to people, canine U_SG_ and U_osm_ have been reported to have high variability between healthy individual dogs, ranging from very dilute (U_SG_ 1.006 g/mL; U_osm_ 273 mOsm/kg) to very concentrated [U_SG_ >1.050 g/mL; U_osm_ 2,600 mOsm/kg; (4, 5)]. Presumably because of the many hormonal and environmental factors, but also possibly because of the day to day variability of free water intake. Therefore, further evaluation on the relationship between daily water intake and urine concentration may help partially explain the high variation in urine concentration, and possibly enable a better estimation of an individual dog's daily water needs and hydration status to maintain “healthy” hydration, particularly in dogs that may be fed once daily and may have a low thirst drive.

The challenge of better understanding optimal daily water needs is not unique to just dogs, as a great deal of research is ongoing in people to establish normative values and acceptable ranges of daily water intake volumes for defining optimal hydration and refining dietary water intake recommendations (6–9). More recent research in people has revealed that subtle shifts in hydration, in particular very mild levels of dehydration (<2% loss of body water), have been associated with both cognitive and exercise performance implications. While these minor changes may seemingly be considered not clinically significant or inconsequential, studies with young adults (men and women) and children indicated that dehydration of <2% loss of BW resulted in impaired cognitive performance and mood (10–12), and dehydrated cyclists with as little as 1% loss of BW had decreased exercise performance (13). Related to this is a recent study of exercise-conditioned search and rescue dogs that were considered adequately hydrated prior to exercise, revealed that skin turgor in the field is sensitive and effective at detecting very mild, acute dehydration (0.8% loss of BW) after only performing 15 min of exercise (14). It is possible that mild dehydration may also have similar effects on cognition and performance for working dogs, as observed in people. Dogs are relied upon to perform lifesaving tasks and working functions in a variety of environmental conditions that include police and military patrol, drug detection, explosives detection, search and rescue, hunting and sporting, biomedical detection services, and various other performance and work activities. All of these tasks invariably rely on high functioning cognitive and physical performance, but also result in loss of hydration. Therefore, a great need exists and additional research is warranted to better establish normative and enahanced water intake levels that should lead to optimally supporting the hydration and water needs of both pet and working dogs while at rest, as well as before, during, and after exercise-related working tasks to ensure optimal health and performance.

Even with daily routines that range from sedentary to a moderately active lifestyle, hydration state is not static during the day. Like people, many situations exist in the pet's daily routine and lifestyle, or as a result of a health condition, that can cause them to trend into hypo-hydration status. Urine osmolality and serum osmolality are sensitive to acute shifts in dehydration, in which acute increases in osmolality can be observed in minutes to hours. However, by evaluating serial urine osmolality measurements over time in an individual animal at rest, it is possible to index the relative chronic hydration status over weeks to months. The link between inadequate hydration and health-related outcomes has been described for a variety of conditions in both people (9) and cats (15–17), including hyperglycemia and accelerated progression of diabetes, higher risk of chronic kidney disease, LUTD, recurrence of kidney stones, and possibly contribute to hypertension. Since these conditions are also prevalent in dogs, inadequate hydration or low daily water intake may also be a risk factor, but more work in these areas is needed.

There is a considerable amount of existing literature in the human nutrition and exercise field associated with the use of water supplements containing organic osmolytes to address rehydration, hydration status, and/or thermoregulation. Some of this research has been recently summarized in several review articles (18–20). Among the various organic osmolytes, glycerol has received significant scientific attention as a water supplement-nutrient for hyperhydration in people, as it has been shown to support improved water retention during post-exercise rehydration (21). In addition, preliminary evidence has been reported in its ability to support hyperhydration in exercising dogs (22). Other nutrition studies have indicated that amino acids in the form of small peptides (23) or whole protein sources from milk (19) can help offset hydration stress when examined using an exercise model in people. Only a few nutrition studies have examined the use of a water supplement to influence hydration or physiological measures in dogs (22, 24, 25). Evaluation of an electrolyte-enriched solution did not demonstrate a benefit to reduce post-exercise body temperature or improve hydration compared to tap water alone (24, 25), whereas the use of a glycerol solution reduced exercise-related dehydration (22).

A greater understanding of normative daily water intake patterns, water balance, and urine indices of chronic hydration status in healthy canines would be valuable. The first objective of the study was to evaluate voluntary tap water (TW) consumption and simultaneously characterize multiple urine and serum measures of chronic hydration. The second objective was to evaluate changes in liquid consumption, total water intake, and urine or serum hydration variables after providing a nutrient-enriched water (NW) as a supplement with free access to TW for 56 days. It is hypothesized that the organic osmolytes and flavor of the NW would enhance liquid intake and improve indices of chronic hydration. Ultimately, the two key goals of this work were to first, model the relationship between daily water intake and corresponding urine concentration/biomarkers. This may lead to future estimations of hydration status, as well as effective daily water requirement estimates that go beyond a generic recommendation of “always provide free access to fresh water.” Second, to evaluate if liquid intake is increased and sustained (7 weeks) when the NW supplement is offered at a moderately increased dose (50% increase in individual daily water requirement) or increased over a short-term (5 days) duration with a higher dose at 2x the individual water requirement. The nutrients in the prototype NW primarily included the organic osmolytes glycerol and amino acids from whey protein and hydrolyzed poultry protein (animal digest).

## Materials and Methods

### Animal Care, Housing, Experimental Design, and Feeding and Watering Regimen

The study protocol was approved by the Nestlé Purina PetCare Animal Care and Use Advisory Committee, followed in strict accordance with the guidelines established by the Nestlé Purina PetCare Animal Care and Use Advisory Committee, and performed at the Nestle Purina PetCare facilities. All dogs used in the trial, prior to pre-trial selection, were evaluated by a veterinarian and determined to be in overall good general health, which included routine serum biochemical analysis. Dogs were housed individually indoors in pens (1.5 × 4.5 m) in a climate controlled facility (ambient temperature ~22°C) with free access to outdoor runs (3.0 × 5.0 m) and exposure to natural light cycles. All dogs were housed at the same kennel location and could see other dogs in adjacent pens. Dogs had direct interaction and socialization with caretakers on a daily basis, which included leash walks outside, and had continuous access to multiple toys.

**Figure 1:**
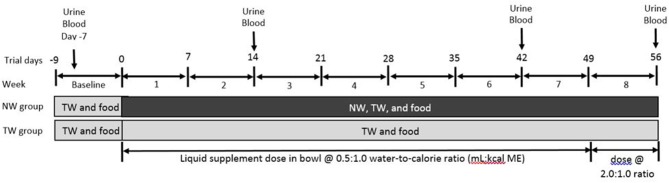
Timeline depicting feeding and watering protocols and sample collection times in a study to evaluate the effects of a nutrient-enriched water (NW) on water intake and indices of hydration in healthy Beagle dogs fed a dry kibble maintanence diet. Dogs were assigned to tap water (TW; *n* = 8) or NW (8) groups following screening during baseline for urine specific gravity ≥1.015; all dogs received TW in a bucket for drinking and had food and TW intake measured during the week before the treatment period (i.e., baseline). Throughout the treatment phase, no changes were made to the food and bucket water regimen for dogs of the TW group except that they also received a dose of TW in a bowl twice daily to coincide with NW dose received by the NW group. Dogs of the NW group were offered NW in a separate bowl to determine water preferences. NW or TW dose for each dog was 0.5:1 or 2:1 water-to-calorie ratio (mL:kcal ME/d) from days 1–49 or 50–56, respectively, based on baseline calorie intake. Measurements were made by manually weigh the bucket and bowl multiple times a day; therefore, to allow for measurements of both water types. Food was provided in a separate bowl for both groups.

The study was designed to monitor individual liquid (grams of liquid) and food intake on a daily basis for a 9-d baseline followed by a 56-d treatment phase (Figure 1). The results section includes both liquid consumption as *free liquid* intake, as well as actual total *water intake* calculated from all 3 water intake sources, which is described in detail below. All dogs had *ad libitum* access to TW in a bucket throughout the entire study and the amount of TW ingested was recorded as grams of water weight three times daily. Total liquid intake (TLI; g/d) was calculated as the sum of test liquid in a bowl and TW in the bucket.

All dogs were fed once daily to maintain body weight with a chicken and rice dry kibble food formulated to meet adult maintenance nutrient requirements (Nestlé Purina PetCare, St. Louis, MO). Proximate analysis (NP Analytical Laboratories, St. Louis, MO) of the diet indicated 5.9% moisture, 28.1% crude protein (AF basis), 17.2% crude fat (AF basis), 1.4% crude fiber (AF basis), 6.5% ash (AF basis). The calculated metabolizable energy of the diet (3,877 kcal/kg) and NW were determined using the modified Atwaters coefficients; 3.5, 8.5, 3.5 for protein, fat, nitrogen free extract (calculated carbohydrates), respectively.

Adult, small breed dogs (*N* = 21) ranging in BW from 7 to 14 kg and age from 2 to 11 years were initially selected for the study. The baseline phase was used to screen the 21 dogs based on their urine specific gravity (U_SG_). Dogs had urine collected by free catch on the morning of day −7, and dogs that had U_SG_ with ≥1.015 were selected for the treatment phase. An unpublished pilot study by our lab revealed that healthy dogs with low U_SG_ (<1.015 g/mL) will exhibit minimal additional reduction in U_SG_ even with greater daily liquid ingestion, and were considered adequately hydrated. Therefore, these dogs were not included in the study population. More importantly, pre-screening of U_SG_ enabled the treatment groups to be balanced prior to starting the treatment phase, as the pilot study also demonstrated that random allocation of dogs based on simply age and sex can result in significantly unbalance U_SG_ between treatment groups. Sixteen dogs (8 males and 8 females) were included for the treatment phase and had an overall average age of 5.6 ± 3.7 years, average weight of 11.7 ± 2.0 kg SD, and body condition score of 5 or 6 on a scale of 1–9 (26).

For the treatment group allocation, initially the selected dogs (*N* = 16) were ranked from highest to lowest U_SG_, and then all odd and even numbered dogs were indicated as group 1 or 2, respectively. These two groups had similar ranges and group mean for U_SG_ (**Table 3**). The TW group was randomly assigned using an online random number generator (www.random.org), consequently the other group was assigned the NW group. The TW or NW were offered in a food bowl twice daily in the a.m. between 08:00 and 10:00 and p.m. between 15:00 and 16:00. The volume of TW or NW for days 1–49 of the treatment phase was based on the calculated water:calorie ratio of 0.5:1 mL/ME kcal. This daily volume was equally divided to be offered in the a.m. and p.m., thus each dog was offered 50% of its daily estimated water requirement based on a conservative requirement of 1:1 mL/ME kcal (1). The test water dose was increased to 2:1 mL/ME kcal for days 50–55 to be representative of a higher dose for a shorter duration of access to the NW with the volume equally divided for a.m. and p.m. administration. As mentioned above, TW in a separate water bucket was available *ad libitum* throughout the entire trial for all dogs, with the exception of when dogs were offered the test water in the food bowl, which represents two of the times during the day when bucket water intake was measured for weight change recorded to the nearest gram.

### Calculation of Total Daily Water Intake

Total water ingestion was calculated for each day and included free water, metabolic water, and food moisture. Free water (g) was either TW (g) or the water-only (g) component of the NW water (grams of dry matter content removed). Metabolic water was calculated based on a conservative estimate of 10 mL water per 100 kcal of ME (27). In addition, metabolic water was calculated for nutrient substrate oxidation of the protein component ingested from the NW water [41 g water per 100 g protein oxidized, (1)] as determined by proximate analysis (Table 1). The individual animal's total daily water intake was calculated based on the total mL of water ingested relative to the total calories (ME kcal) ingested on a daily basis (water:calorie ratio), as well as total mL of water per kg BW per day (mL/kg BW).

**Table 1:** Ingredient composition, proximates, mineral content, and calorie content of a nutrient-enriched water ingested by sedentary dogs at rest for 56 days to evaluate water intake and indices of hydration.

	**Nutrient-enriched Water**
**INGREDIENTS (%)**	
Whey protein	2.4
Glycerin	1.0
Potassium chloride	0.1
Poultry digest	1.0
Water	95.5
**FINISHED PRODUCT ANALYSES**	
Moisture	95.9
Crude protein (% as fed)	2.7
Crude fat (% as fed)	0.27
Crude fiber (% as fed)	<0.2
Ash (% as fed)	<0.2
Phosphorus (% as fed)	0.018
Potassium (% as fed)	0.064
Sodium (% as fed [mEq/L])	0.039 [16.9]
Calculated ME (kcal/100mL)	14.3

### Sample Collection and Analysis

To evaluate various physiological parameters associated with hydration, overnight fasted blood and urine samples of free-catch voided urine were collected in the morning between 07:00 and 09:00 on days −7, 14, 42, and 56 (Figure 1). Urine samples were analyzed on the day of collection for specific gravity with a refractometer (HSK-VET, Veterinary refractometer, Heska, Loveland, Colorado), osmolality (Vapro 5520 Vapor Pressure Osmometer, Wescor Inc, Logan, Utah), pH (pH 11 Meter, Oakton Instruments, Vernon Hills, Illinois), urea nitrogen, and creatinine by means of an automated biochemical assay system (Cobas, model c311, Roche Diagnostics, Indianapolis, Indiana). Stored urine samples (−80°C) were also analyzed for Na^+^ using inductively coupled plasma optical emission spectroscopy (ICP-OES, Perkin Elmer Optima™ 2,000 DV, PerkinElmer, Inc., Shelton, CT) and phosphate using ion chromatography (ICS−5000, Dionex Inc., Sunnyvale, California).

Venous blood was transferred to blood tubes (BD Vacutainer SST tubes, Becton, Dickinson and Company, Franklin Lakes, NJ) and allowed to clot for 10 min at room temperature. Serum was collected after centrifugation of clotted blood samples and stored at −80°C until aliquoted samples were analyzed for osmolality using an osmometer^d^ and clinical chemistry profiles by means of an automated biochemical assay system (Cobas, model c311, Roche Diagnostics, Indianapolis, Indiana) in accordance with the manufacturer's instructions. Clinical chemistry analytes were assayed using Cobas kits from the system manufacturer (Roche Diagnostics, Indianapolis, Indiana) included albumin, alanine aminotransferase, creatine kinase, creatinine (enzyme method), gamma-glutamyl transferase, glucose, phosphorus, potassium, sodium, and urea nitrogen.

### Statistics

A linear mixed-effects model was used to account for the nonindependence of the data with a commercially available R lme4 software package [R Core Team (2015). R: a language and environment for statistical computing, version 3.2.1. Foundation for Statistical Computing, Vienna, Austria. Available at: www.r-project.org. Accessed 12-07-2017; (28)]. Dog identification was used as a random effect, and the intercept was allowed to vary by dog. Treatment (TW vs. NW), time, and the interaction between treatment and time were entered as fixed effects. Satterthwaite approximation of degrees of freedom were used to calculate the *P*-values. Tukey *post-hoc* tests were then conducted. In addition to examining the linear relationship between U_SG_, U_osm_, S_osm_, and liquid intake, quadratic polynomial models were also examined. To determine if adding the quadratic term improved model fit, two models were run—one model without and one model with the quadratic term. The fit of the two models were then examined using the likelihood ratio tests to determine if adding the quadratic term resulted in improved model fit. Analyses were considered to be significant at alpha = 0.05. Previous pilot studies indicated that *N* = 12 dogs per treatment group would result in a statistical power of 0.81 to achieve statistical significance at alpha = 0.05 and an effect size of 0.9 based on urine osmolality data.

With the current study data, power analysis based on a two-sample *t*-test (2 sided equality) using urine specific gravity data resulted in a statistical power of 0.81 to achieve statistical significance at alpha = 0.05 when treatment means differ by 0.016 g/mL with *N* = 8 dogs per treatment group. Alternatively for total daily water intake data, a statistical power of 0.80 to achieve statistical significance at alpha = 0.05 required treatment means to differ by 600 mL per day with *N* = 8 dogs per treatment group.

## Results

### Food Calorie Intake, Free Liquid Drinking, and Body Weight

**Figure 2:**
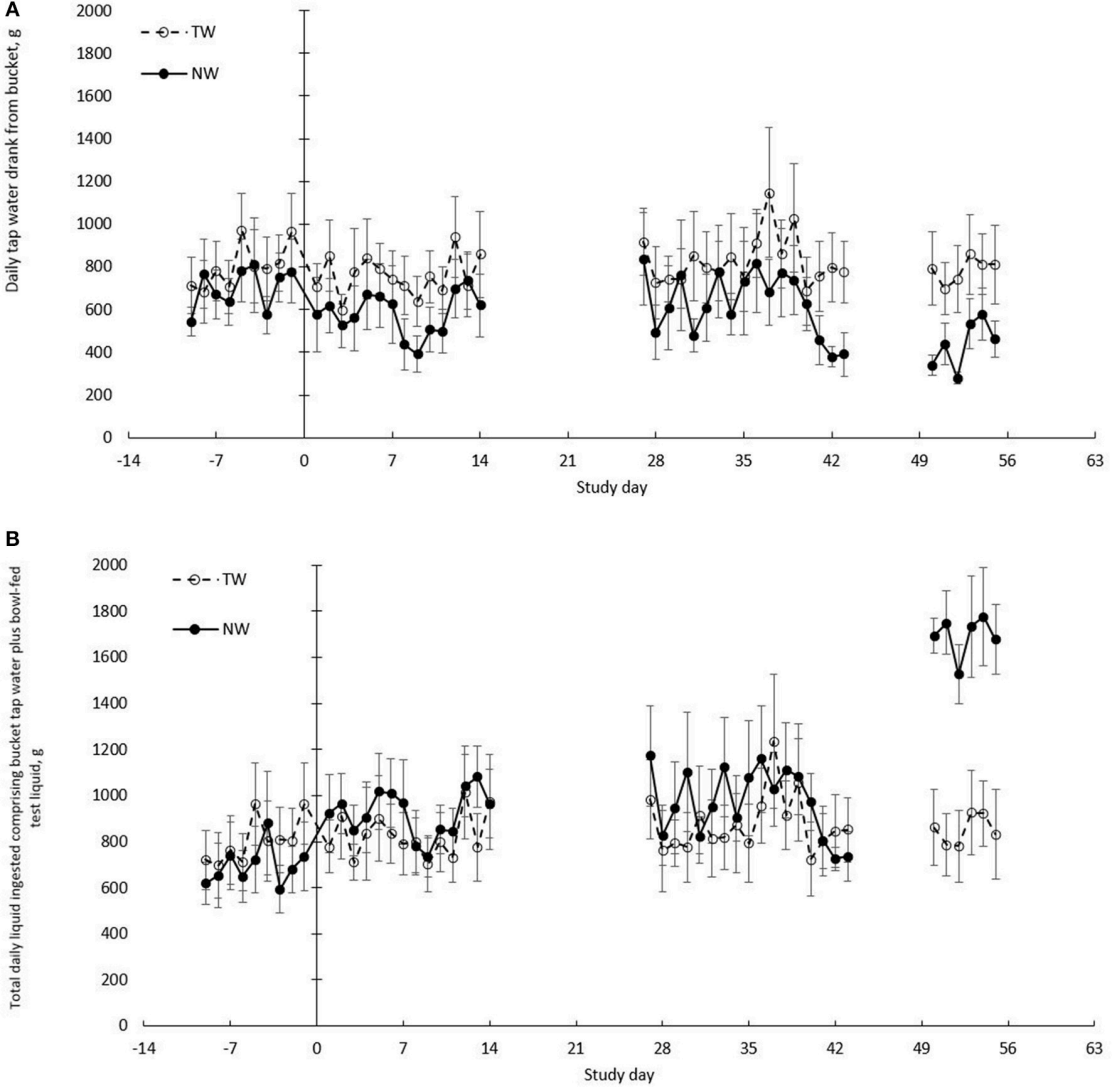
Daily total free liquid drank in healthy dogs. **(A)** Mean (±SE) daily bucket tap water volume (TW) drank (g/d) when fed dry food and *ad libitum* tap water offered in a bucket. **(B)** Mean (±SE) daily total liquid volume drank (g/d) calculated as the sum of total daily bucket TW and daily TW or nutrient-enriched water (NW) dose offered in a bowl. Liquid treatment groups included TW (*n* = 8) or a NW (*n* = 8). All treatment groups received only tap water in a bucket during baseline days −9 to −1. Treatment phase included all dogs having *ad libitum* bucket TW with the NW group having access to a specific dose of NW in a bowl twice daily and the TW group having access to a specific dose of TW in a bowl twice daily.

Daily liquid (bowl and bucket) and food calorie intake during the baseline week and weeks 1, 2, 5, 6, and 8 of the treatment phase were measured. Daily liquid intake was not recorded during weeks 3, 4, and 7. Each dog's daily liquid intake was provided as TW drank from a bucket, as well as from a bowl containing the test liquid dose (TW or NW). Daily drinking patterns of bucket TW intake (g/d; Figure 2A) and total liquid intake (g/d; bucket TW plus test liquid in a bowl; Figure 2B) are plotted over the entire duration of the trial to graphically illustrate the daily variation of each treatment group.

Weekly liquid and food calorie intake averages were calculated and statistically analyzed to compare within and between groups (Table 2). Mean food calorie intake did not differ between treatment groups (*P* = 0.88) or for time-by-treatment interaction (*P* = 0.49). The effect of time was significant (*P* < 0.001), as mean calorie intake was greater (*P* < 0.01) for all dogs during baseline compared to all subsequent weeks during the treatment phase. Weekly body weight resulted in a significant time x treatment interaction (*P* < 0.001; Table 2), in which BW increased on average by 2% between the beginning and the end of the 56-d trial for the NW group, but declined by 5.6% over the same period of time for the TW group. Pairwise comparisons of BW means between the treatment groups were never different at any time point.

**Table 2:** Mean (±SE) body weight, liquid drank, total water intake, and calorie intake in sedentary adult dogs offered tap water or a nutrient-enriched water, in addition to *ad libitum* access to tap water in a bucket throughout the entire study duration.

	**Baseline**		**Treatment phase**												
	***Ad libitum*** **TW only**		**Daily NW dose @ 0.5:1 water:kcal ratio**								**Daily NW dose @ 2:1 water:kcal ratio**		***p***-values^*^		
**Measures and treatment groups**	**Week 1**		**Week 1**		**Week 2**		**week 5**		**Week 6**		**Week 8**		**Time**	**trt**	**trt x time**
	**Mean**	**SE**	**Mean**	**SE**	**Mean**	**SE**	**Mean**	**SE**	**Mean**	**SE**	**Mean**	**SE**			
**BODY WEIGHT, KG**															
TW	12.3^a,A^	0.9	12.2^a,A^	0.9	12.1^b,A^	0.9	ND		11.8^c,A^	0.8	11.6^d,A^	0.8	<0.001	0.43	<0.001
NW	11.1^a,A^	0.5	11.2^a,b,A^	0.5	11.2^a,b,A^	0.5	ND		11.2^a,b,A^	0.5	11.3^b,A^	0.5			
**FOOD CALORIE INGESTION, ME KCALS/d**															
TW	717	46	670	41	663	45	684	38	684	38	684	38	<0.001	0.88	0.49
NW	697	68	671	58	671	58	665	52	665	52	665	52			
**TOTAL BUCKET WATER INTAKE, G/D**															
TW	804	147	759	127	758	136	788	148	884	186	786	141	0.01	0.30	0.11
NW	701	124	606	140	555	111	649	162	639	126	438	67			
**TOTAL TEST LIQUID INTAKE, G/D**															
TW	NA		64^a,A^	14	70^a,A^	17	38^a,A^	9	49^a,A^	12	65^a,A^	15	<0.001	<0.001	<0.001
NW	NA		343^a,B^	10	346^a,B^	11	341^a,B^	9	344^a,B^	10	1255^b,B^	104			
**TOTAL LIQUID INTAKE, G/D**															
TW	804^a,A^	147	823^a,A^	133	828^a,A^	139	826^a,A^	151	933^a,A^	183	851^a,A^	140	<0.001	0.34	<0.001
NW	696^a,A^	123	948^b,A^	141	901^a,b,A^	111	990^b,A^	163	982^b,A^	129	1693^c,B^	139			
**TOTAL WATER INTAKE, ML WATER:ME KCAL/D**															
TW	1.22^a,A^	0.18	1.31^a,A^	0.13	1.33^a,A^	0.14	1.29^a,A^	0.16	1.43^a,A^	0.18	1.33^a,A^	0.15	<0.001	0.11	<0.001
NW	1.09^a,A^	0.09	1.50^b,A^	0.07	1.44^b,A^	0.07	1.57^b,A^	0.08	1.55^b,A^	0.08	2.45^c,B^	0.18			
**TOTAL WATER INTAKE, ML/KG BW/D**															
TW	71^a,A^	9	74^a,A^	9	74^a,A^	9	75^a,A^	9	84^a,A^	12	79^a,A^	9	<0.001	0.14	<0.001
NW	71^a,A^	12	92^b,A^	13	87^a,b,A^	11	96^b,A^	16	94^b,A^	12	156^c,^B^^	13			

*TW:tap water. NW: nutrient-enriched water. ME: metabolizable energy*.

a,b *Within a row, values with different lowercase superscript letters differ significantly (pairwise comparisons) within a treatment group*.

A,B *Within a column, values with different uppercase superscript letters differ significantly (pairwise comparisons) between the 2 treatment groups. For all pairwise comparisons, values of p < 0.05 were considered significant*.

* *p values were generated from a linear mixed model*.

In addition, the mean weekly total bucket water did not differ between treatment groups (*P* = 0.30) or for time-by-treatment interaction (*P* = 0.11). The effect of time was significant (*P* = 0.01), as the overall mean for total bucket water of all 16 dogs during week 8 was lower (*P* < 0.03) compared to baseline and week 6, but similar to weeks 1, 2, and 5.

At the start of the treatment phase, test liquid dose was initiated for both groups. From day 1 of week 1 through the end of week 7, the mean daily liquid dose offered in a bowl was 371 or 346 g/d for the TW or NW groups, respectively, based on the daily dose @ 0.5:1 water:kcal ratio (0.5x dose). During week 8 the daily dose was increased to 2:1 water:kcal ratio (2x dose) to assess if the dogs would self-regulate their water intake when either the TW or NW was available in excess in the bowl. The average daily dose was 1,484 or 1,384 g/d, respectively for the TW or NW groups.

All *N* = 8 dogs in the NW group readily accepted and ingested the NW. To summarize, during weeks 1 through 7 when dogs were offered the 0.5x dose, all dogs (*N* = 8) in the NW group drank nearly 100% of the liquid offered in a bowl, whereas all the dogs in the TW group drank between 10 and 20% of the volume offered (Table 2). During week 8 with the 2x dose, the liquid ingested in a bowl for the TW group was similar to previous weeks. By contrast, the mean for the NW group was 91% ingested of total NW offered in a bowl, as all but two dogs in the NW group drank >99% of the NW. Based on the average liquid intake volumes of NW (Table 2), and that the NW was calculated to be 14.3 kcal/100 mL NW (Table 1), the NW group ingested 49 (kcal; 7.4%) additional calories per day from the NW relative to the total daily food calories from days 1–49, and 180 (27%) additional calories per day from days 50–56. Over the entire 8-week treatment phase, TW drank from the bucket declined between 10 and 30% for NW group compared to baseline drinking volume, but the TW group varied by <2%, except for week 6. There was significant for time-by-treatment interaction (*P* < 0.001) for test liquid intake.

Total liquid intake (TLI; g/d) was calculated for each group and reported in Table 2. A time-by-treatment interaction was significant (*P* < 0.001). The mean baseline TLI was similar between treatment groups. For dogs in the TW group, no difference was observed between any of the weeks during the study, and ingested a similar amount of TW during week 8 compared to all previous weeks regardless of the extra TW offered in the bowl. For the NW group, TLI significantly (*P* < 0.05) increased at every measurement week, except for week 2 (*P* = 0.07) compared to baseline. During week 8 with the 2x dose of NW, TLI was significantly (*P* < 0.001) greater than all previous weeks during the treatment phase when dogs were offered the 0.5x dose of NW.

### Total Water Intake and Water-to-Calorie Intake Ratios

Calculated daily total water intake (TWI; the sum of FW consumed by drinking [TW and the water-only component of NW], MW [conversion from food and NW nutrients], and food moisture) was determined and used to calculate mean daily water-to-calorie intake ratios (as mL per kcal ME; Table 2). Daily TWI was also calculated by adjusting for body weight of the dogs (mL/kg). A significant (*P* < 0.001) time-by-treatment interaction was identified for both the water-to-calorie intake ratio and for TWI on a BW basis. At baseline, both water intake measures were similar between the TW and NW groups, and the TW group did not differ between all weeks during the treatment phase. In contrast, water-to-calorie intake ratio was significantly (*P* < 0.05) increased for the NW group during all weeks of the treatment phase relative to baseline. Week 8 was significantly (*P* < 0.001) greater than all other treatment phase weeks. Similarly, daily TWI adjusted for BW also significantly (*P* < 0.008) increased during all weeks except week 2 (*P* = 0.10) compared to baseline.

### Urine and Serum Characteristics

The U_SG_, U_osm_, and pH data were summarized (Table 3). A significant (*P* < 0.002) time-by-treatment interaction was identified for all three measures. Dogs of the TW group had a similar U_SG_, U_osm_, and pH compared to the NW group at baseline. The U_SG_, U_osm_, and pH for the TW group remained generally stable throughout the study, with the U_SG_ group mean varying among time points by ≤ 0.006 g/mL and U_osm_ varying between time points by ≤ 104 mOsm/kg. Dogs of the NW group had a significantly (*P* < 0.01) lower mean U_SG_ on days 42 when were offered the 0.5x dose of NW and day 56 when were offered the 2x dose of NW compared to baseline. In addition, mean U_osm_ for the NW group significantly (*P* < 0.05) decreased on all sampling days of the treatment phase compared with the baseline value.

**Table 3:** Mean (±SE) urine measures intake in sedentary adult dogs (*n* = 16) offered TW or a nutrient-enriched water in addition to *ad libitum* access to TW in a bucket.

	**Baseline**		**Treatment phase**								
	***Ad libitum*** **TW only**		**NW @ 0.5:1 water:kcal ratio**				**NW @ 2:1 water:kcal ratio**		***p***-values^*^		
**Measures and treatment groups**	**Day 7**		**Day 14**		**Day 42**		**Day 56**		**Time**	**trt**	**trt x time**
	**Mean**	**SE**	**Mean**	**SE**	**Mean**	**SE**	**Mean**	**SE**			
**CREATININE, MG/DL**											
TW	138^a,A^	34	179^a,A^	29	195^a,A^	31	185^a,A^	19	0.44	0.009	0.002
NW	135^a,A^	19	110^a,b,A^	14	91^a,b,B^	10	62^b,B^	9			
**pH**											
TW	6.6^a,A^	0.4	6.1^a,A^	0.2	6.0^a,A^	0.3	6.1^a,A^	0.2	0.80	0.47	0.002
NW	5.9^a,A^	0.2	6.4^a,b,A^	0.2	6.5^b,A^	0.3	6.7^b,A^	0.3			
**PHOSPHATE, MMOL/L**											
TW	41	12	16	5	44	8	38	11	0.06	0.15	0.34
NW	62	13	47	12	48	6	38	10			
**USG, G/ML**											
TW	1.025^a,A^	0.004	1.031^a,A^	0.004	1.029^a,A^	0.004	1.030^a,A^	0.004	0.21	0.06	<0.001
NW	1.026^a,A^	0.003	1.021^a,b,A^	0.003	1.018^b,A^	0.002	1.014^b,B^	0.001			
**SODIUM, MMOL/L**											
TW	132	28	73	22	77	22	80	15	0.12	0.70	0.77
NW	114	16	730	16	101	14	87	18			
**U**_**osm**_**, mOsm/kg**											
TW	1024^a,A^	172	1142^a,A^	153	1077^a,A^	160	1128^a,A^	148	0.06	0.12	<0.001
NW	1089^a,A^	152	811^b,A^	102	719^b,A^	92	635^b,A^	67			
**UREA NITROGEN, MG/DL**											
TW	1408^a,A^	334	1810^a,A^	266	1869^a,A^	270	2059^b,A^	296	0.87	0.08	<0.001
NW	1686^a,A^	274	1296^a,b,A^	179	1072^a,b,A^	124	875^b,B^	102			

*TW: tap water. NW: nutrient-enriched water*.

a,b *Within a row, values with different lowercase superscript letters differ significantly (pairwise comparisons) within a treatment group*.

A,B *Within a column, values with different uppercase superscript letters differ significantly (pairwise comparisons) between the 2 treatment groups. For all pairwise comparisons, values of p < 0.05 were considered significant*.

* *p values were generated from a linear mixed model*.

A significant (*P* = 0.002) time-by-treatment interaction was observed for urine pH. Specifically, mean urine pH did not differ between groups at baseline or any other sampling time point. For the TW group, the pH did not differ between sampling times. By contrast, mean baseline pH was lowest for the NW group and differed (*P* ≤ 0.05) compared to days 42 and 56. Urine analytes, creatinine and urea nitrogen, resulted in a significant (*P* ≤ 0.020) time-by-treatment interactions (Table 3). Mean concentration of both analytes were similar between treatment groups at baseline. For the TW group, mean creatinine was not different between all sampling times, but urea nitrogen was significantly (*P* < 0.05) higher on day 56 compared to all previous sampling days. The NW group had a significant (*P* < 0.05) decrease in mean urine creatinine and urea nitrogen concentration at day 56 when the NW was offered at 2x dose compared to baseline, but was similar to days 14 and 42 when the NW dose was 0.5x. No significant main effects of time, treatment, or time-by-treatment interaction were observed for urine phosphate and sodium.

The serum analyte concentration data are reported in Table 4. Time-by-treatment interactions were not significant (*P* ≥ 0.12) for any serum measure except for urea nitrogen (*P* < 0.001). No significant main effect of treatment was observed, except for sodium (*P* < 0.04). Several analytes were significant (*P* ≤ 0.02) for the main effect of time, including creatine kinase, creatinine, glucose, S_osm_, potassium, and sodium.

**Table 4:** Mean (±SE) serum measures in sedentary adult dogs (*n* = 16) offered TW or a nutrient-enriched water during trial 1 in addition to *ad libitum* access to TW in a bucket.

	**Baseline**		**Treatment phase**								
	***Ad libitum*** **TW only**		**NW @ 0.5:1 water:kcal ratio**				**NW @ 2:1 water:kcal ratio**		***p***-values^*^		
**Measures and treatment groups**	**Day 7**		**Day 14**		**Day 42**		**Day 56**		**Time**	**Trt**	**Trt x time**
	**Mean**	**SE**	**Mean**	**SE**	**Mean**	**SE**	**Mean**	**SE**			
**ALBUMIN, G/DL**											
TW	3.8	0.1	3.9	0.1	3.7	0.1	3.8	0.1	0.21	0.89	0.12
NW	3.8	0.1	3.8	0.1	3.8	0.1	3.9	0.1			
**ALT, U/L**											
TW	30.6	2.7	30.4	2.4	29.5	3.9	67.5	35.2	0.39	0.87	0.41
NW	39.5	5.3	44.6	12.7	41.4	9.5	42.2	6.5			
**CREATINE KINASE, U/L**											
TW	148	15	150	21	109	12	156	16	0.02	0.16	0.63
NW	195	32	198	29	160	22	196	26			
**CREATININE, MG/DL**											
TW	0.73	0.04	0.71	0.05	0.67	0.03	0.64	0.03	0.007	0.22	0.56
NW	0.64	0.04	0.63	0.03	0.65	0.03	0.59	0.04			
**GGT, g/dL**											
TW	3.5	0.2	2.5	0.4	3.7	0.3	3.9	0.9	0.67	0.27	0.12
NW	5.3	0.4	4.7	0.7	4.2	1.0	3.5	0.6			
**GLUCOSE, MG/DL**											
TW	94	2	105	2	103	2	102	2	<0.001	0.58	0.29
NW	94	3	100	2	104	2	105	2			
**S**_**osm**_**, mOsm/kg**											
TW	309	2	310	2	303	2	306	2	<0.001	0.22	0.65
NW	314	1	310	1	304	2	307	2			
**PHOSPHORUS, mg/dL**											
TW	4.1	0.1	4.0	0.1	4.2	0.2	4.0	0.2	0.20	0.99	0.33
NW	4.1	0.1	3.7	0.2	3.9	0.1	4.0	0.2			
**POTASSIUM, MMOL/L**											
TW	4.5	0.1	4.4	0.1	4.4	0.1	4.4	0.2	0.006	0.06	0.38
NW	4.7	0.1	4.4	0.1	4.5	0.1	4.7	0.2			
**SODIUM, MMOL/L**											
TW	150	1	145	4	150	1	151	1	0.01	0.04	0.52
NW	151	1	151	1	152	1	154	1			
**UREA NITROGEN, MG/DL**											
TW	15.0^a,A^	1.0	14.1^a,A^	1.0	13.7^a,A^	0.8	13.5^a,A^	0.9	0.006	0.12	<0.001
NW	14.8^a,A^	0.7	14.3^a,A^	0.6	15.0^a,b,A^	0.5	16.9^b,B^	0.5			

*TW:tap water. NW: nutrient-enriched water*.

a,b *Within a row, values between time points with different lowercase superscript letters differ significantly (pairwise comparisons) within a treatment group*.

A,B *Within a column, values between the 2 treatment groups with different uppercase superscript letters differ significantly (pairwise comparisons). For all pairwise comparisons, values of p < 0.05 were considered significant*.

* *p values were generated from a linear mixed model*.

### Relationships Among Measures of Hydration and Liquid Intake

**Figure 3:**
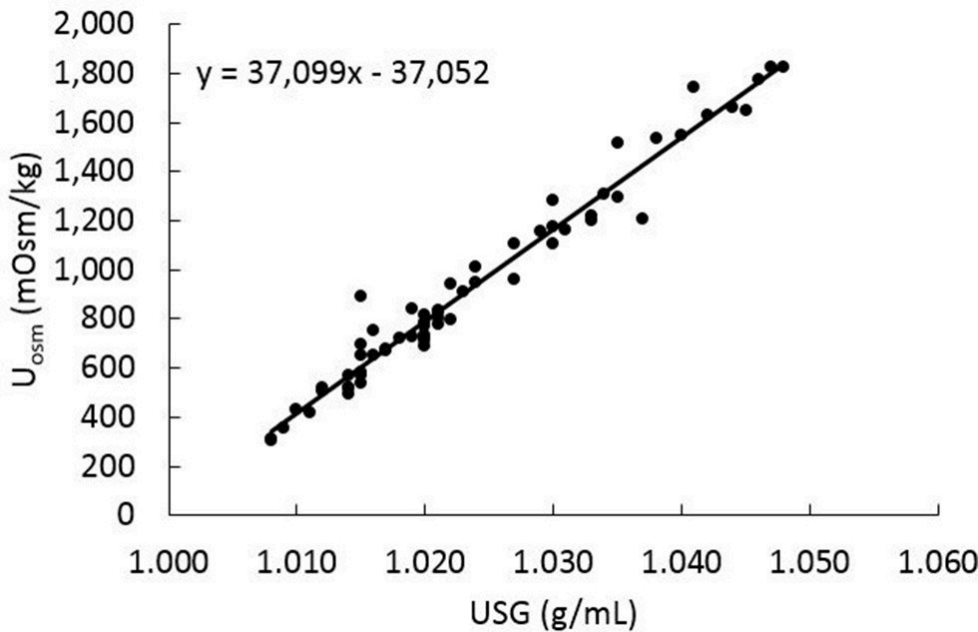
Results of linear mixed-effects model analysis for urine osmolality vs. specific gravity for the same 16 dogs as in Figure 1. Each data point represent each dog at each sample collection time (days −7, 14, 42, and 56).

**Figure 4:**
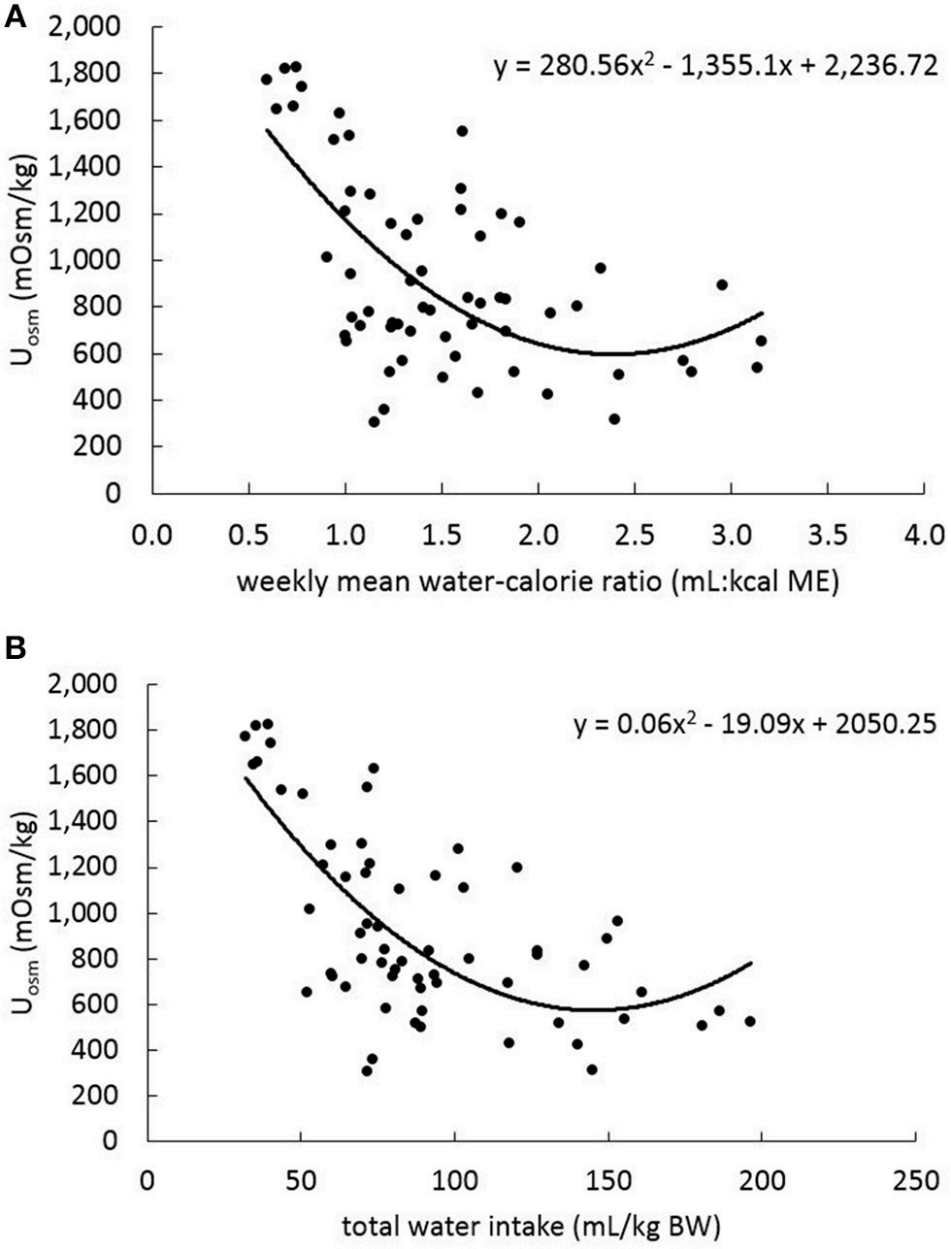
Results of mixed effects regression analysis for relationships between urine osmolality (U_osm_; days −7, 14, 42, and 56) and water intake variables for the same 16 dogs as in Figure 1. The U_osm_ vs. weekly mean **(A)** water-to-calorie intake ratios and **(B)** total water intake on a BW basis (determined on the basis of calculated total water intake, which included FW [TW and the water-only component of NW], MW [conversion from food and NW nutrients], and food moisture components) during baseline week, week 2, week 6, and week 8. **(A, B)** include data points that each represent each dog at each sample collection time.

In the linear mixed-effects model (with data from all sample collection periods), U_SG_ and U_osm_ were positively and significantly (*P* < 0.001) related with each other (β = 37098.77; Figure 3). To evaluate how water consumption related to changes in U_osm_, the weekly water-to-calorie intake ratio data from week −1, 2, 5, and 7 were compared with U_osm_ data from day −7 (baseline) and days 14, 42, and 56 (treatment phase) by mixed-effects model analysis. A significant negative relationship (β = −265.25; *P* < 0.001) was observed, however, a second-order polynomial model fit the data better than the linear model (χ^2^ = 11.33, *P* < 0.001: Figure 4). The relationship between U_osm_ and total water intake adjusted for body weight was also evaluated and resulted in an negative linear relationship (β = −4.89; *P* < 0.001), and a second-order polynomial model fit the data better than a linear model (χ^2^ = 9.63, *P* < 0.01: Figure 4). A maximum dilution of urine (i.e., minimum U_osm_) with increasing amounts of liquid ingestion appeared to occur at ~300 mOsm/kg.

## Discussion

Daily water needs in healthy dogs are not well defined and little is known regarding how incremental changes in water intake translate into changes in blood or urine measures associated with hydration. The 2 objectives of the present study were achieved, first by further characterizing how daily water drinking corresponds to changes in several variables associated with urine indices of hydration in healthy dogs eating dry food. Second, the study results revealed that the NW treatment in dogs with U_SG_ >1.015 was significantly associated with changes in the amount of water consumption that resulted in increased total water intake and dilution of urine. This study generated several unique findings, which included a significantly greater preference for consumption of the NW by the dogs when free access to TW was also available, and that high liquid intake helped maintain a more dilute urine over a 2-month study period. The study also generated evidence of a curvilinear model for water intake that appears necessary to dilute urine concentration for healthy dogs eating dry food, although further research is needed to confirm this.

Mean daily TW and dry food ingestion for dogs in TW and NW groups during baseline were similar, with TW intake adjusted for body weight varying only slightly on a daily basis (range, mean ± SD; 60 to 82; mean 71 ± 8 mL/kg/d). Over the entire study for the TW group, the weekly mean total water intake (determined on the basis of total water intake [ie, FW consumed by drinking, MW, and food moisture components]) and adjusted for BW was consistent with previously reported mean of ~63–73 mL/kg/d for dogs drinking TW and fed dry food (29, 30). However, dogs drinking the NW had a significant (*P* < 0.001) increase in mean total water intake from 71 mL/kg/d during baseline to at least 92 mL/kg/d when offered the 0.5x dose of NW, and approximately doubled to 156 mL/kg/d when provided the NW at the 2.0x dose.

Another method of reporting daily total water intake is to adjust based on the daily calorie intake to determine the water-to-calorie intake ratio. Although not readily reported, the water-to-calorie intake ratio of 1.0:1.0 mL/kcal ME is suggested as a conservative estimate of a dog's minimum water requirement in the 2006 NRC chapter on water (1). Our study reports a slightly higher weekly mean ratio for the TW group that ranges from 1.2:1.0 to 1.4:1.0 mL/kcal ME in this group of dogs that sustained a group mean USG between 1.025 to 1.030 g/mL. This suggests that the conservative estimate of 1.0:1.0 mL/kcal ME underestimates their water requirement, particularly since the conservative estimate references are generalizations from book chapters (31, 32) and not specific peer-reviewed research reports. Importantly, the method of measuring water-to-calorie intake ratio was also sensitive to detect changes in total water intake in dogs drinking the NW, as they had a significant (*P* < 0.001) increase in mean water-to-calorie intake ratio of 1.1:1.0 mL/kcal ME during baseline to at least 1.4:1.0 mL/kcal ME when offered the 0.5x dose of NW.

The NW used in the present study contains ingredients that supply various nutrients that are considered osmolytes, mostly amino acids, glycerol, and electrolytes. Total solids in the NW are calculated to be ~4.37% by summation of the proximate analysis results on an as-fed basis, which includes 1% glycerol not measured by proximate analysis. The solids portion of the NW formula was largely composed of whey protein concentrate and a hydrolyzed poultry digest in addition to glycerol. This was confirmed with the proximate analysis that the NW contained 2.7% crude protein. The NW was also determined to contain 0.039% sodium and 0.018% phosphorus. On the basis of mean NW consumption measured from week 1 through 6 (~343 g/dog/d), dogs in the NW group consumed a mean of ~133 mg of sodium and 61.7 mg of phosphorus/d as ingested by drinking the NW. Although electrolytes are present in the formula, they are a much smaller proportion of osmolytes (0.057%) when compared with the amino acids (2.7%) and glycerol (1%). Thus, it is likely that the significant increase in liquid drinking by dogs of the NW group was primarily attributable to the whey and glycerol, as well as poultry flavor, and not the low sodium content. Additional work is necessary to isolate the effects of these separate nutritional components on liquid intake.

Evidence of urine-based biomarkers of hydration for people has been growing in the past 5 years, such that U_SG_, U_osm_, and urine color have demonstrated significant promise in estimating water intake in healthy adults (10, 33–38). The current study was designed to collect 4 urine samples over a 2-month study period for measurement of these same urine parameters and determine if these urine measures in healthy adult dogs could also facilitate an estimate of water intake. For dogs included in the TW group, these periodic assessments of urine biomarkers of hydration revealed that the mean concentrations of urine analytes and urine density from samples collected free catch first thing in the morning remained relatively unchanged, as all of these dogs had U_SG_ and U_osm_ measurements within a fairly narrow range throughout the study. From our study, the overall mean U_SG_ of 1.029 g/mL and U_osm_ of 1,093 mOsm/kg were slightly lower compared to U_SG_ [1.035-1.040 g/mL; (4, 39)] and U_osm_ [1100–1500 mOsm/kg; (4, 5, 39)] values reported for healthy dogs in other studies. However, one consistency existed between our study and other reported data, such that there can be high variability of urine concentration between healthy individual dogs ranging from very dilute (U_SG_ 1.006 g/mL; U_osm_ 273 mOsm/kg) to very concentrated [U_SG_ >1.050 g/mL; U_osm_ 2,600 mOsm/kg; (4, 39)]. This natural variance became particularly evident following the completion of an unpublished initial pilot study by our lab that revealed that randomly assigning healthy dogs to treatment groups without analyzing and prescreening for U_SG_ or U_osm_ can easily result in significantly unbalanced treatment groups at baseline based on U_SG_ or U_osm_, thus making changes on urine dilution difficult when the dog starts out with a very low U_SG_.

Ultimately, this pilot study was the basis for collection of an initial urine sample during baseline of the current study for pre-screening and balancing of treatment groups prior to starting the treatment phase. Furthermore, while a potential bias, the unpublished pilot study also revealed that healthy dogs with low U_SG_ (<1.015 g/mL) will exhibit minimal further reduction in U_SG_ even with greater daily liquid ingestion. While it is important to consider this sub-group of dogs, in general they are either adequately hydrated because of sufficient water intake or potentially at risk of being unable to concentrate their urine. In this current study, all dogs, including those with U_SG_ < 1.015 g/mL, were determined to be healthy based on pre-trial clinical chemistry and urinalysis assessment by the attending veterinarians, but were excluded from the study as part of the screening procedure.

Ingestion of the NW in this study was significantly associated with changes in urine variables that are interpreted as improved hydration, including decreased U_SG_, decreased U_osm_, and lower concentrations of some urine analytes, but not minerals (sodium and phosphate), relative to the baseline data. It is notable that the dogs receiving the NW had significant reductions in mean urine concentrations of creatinine, and urea nitrogen, which appears attributable to more dilute urine during the treatment period. As stated above, this study would have been improved with the inclusion of urine void volume data to complement the urine analyte concentrations and density, and would contribute to a recently proposed alternative approach to assess hydration in people as a “process,” instead of a “state” (9).

Urine osmolality is routinely reported in studies related to hydration status and our data has confirmed that it was highly correlated with U_SG_ in dogs (5) and in cats (40). The linear mixed-effects model analysis of total water-to-calorie intake ratios and U_osm_ determined with all 16 dogs revealed a significant (*P* < 0.001) inverse relationship between these variables. This polynomial model is similar to previously reported data with 35 dogs^2^, which predicted that a U_osm_ of 1,000 mOsm/kg would result from total water intake calculated as water-to-calorie intake ratio of ~1.1 mL: 1 kcal ME. The polynomial model reported in this current study, based on also using 1,000 mOsm/kg, would estimate the water-to-calorie intake ratio of ~1.2 mL: 1 kcal ME. Alternatively, evaluation of the total water intake on a BW basis also revealed a significant (*P* < 0.001) second-order polynomial model. These relationships enable U_osm_ data to estimate a healthy dog's daily water intake in the absence of a pet owner attempting to measure actual water consumption volume. Although the number of dogs in the study was small, with only 8 dogs receiving the NW, further research is needed to confirm these findings. Because of the very high relationship between U_SG_ and U_osmo_, we would expect and observed (data not shown) that U_SG_ was also significantly and similarly related to total water intake measures.

In conclusion, our findings indicated that healthy dogs can have greater total water intake and improved indices associated with chronic hydration when a palatable NW is supplied daily for drinking. Therefore, a water supplement appears to be a feasible alternative method to increase water ingestion in dogs. Since this NW composition also contains calories from the nutrients, additional calorie content did occur as a result of the volume consumed. However, ingestion of the NW at the 0.5x dose over the 7 week study was estimated to provide 7.4% of additional daily calories, which is below the generally accepted level of 10% additional calories recommended for offering daily treats to pet dogs, and no change in BW was observed during the study. Adminstration and ingestion of the 2x dose provided considerably greater calorie intake during the 5 day feeding period and prolonged ingestion of the 2x dose to non-exercising dogs may contribute to excess weight gain similar to ingestion of treats in excess of 10% of daily calorie requirement. Although out of scope for this study and data, this greater liquid portion would likely be more appropriate for brief applications related to re-hydration in very active working dogs where daily calorie intake is likely higher (thus the NW is a lower percentage of total calories), as well as when calorie expenditure and water loss are elevated because of exercise. More studies are necessary to explore this application.

Ultimately, the present study provided a basis for greater understanding of water needs in healthy dogs and how the amount of daily water ingestion impacts various measures of hydration and physiologic variables. One of the strengths of this study is the demonstration that increased liquid intake can be sustained over a period of multiple consecutive weeks. Furthermore, that this study reports data associated with chronic hydration in dogs ingesting simply tapwater, as well as dogs ingesting the water supplement that demonstrated greater and chronic level of hydration. Going forward, research to demonstrate specific benefits related to minimizing dehydration for highly active or exercising dogs, or support for sick or injured dogs, is necessary and warranted based on the current findings in healthy dogs. Many situations or health conditions can arise that cause hypohydration in pets. Based on this work and use of a water supplement to facilitate a greater level of voluntary liquid intake in dogs, additional studies can be explored for dogs that need additional veterinary support to lower the risk of urolith formation or require management for renal insufficiency, LUTD, or hypohydration resulting from age, injury, or surgery.

## Author Contributions

All authors listed have made a substantial, direct and intellectual contribution to the work, and approved it for publication.

### Conflict of Interest Statement

BZ and CG are employed within the R&D department of Nestlé Purina PetCare and conduct nutrition research for the potential use in future commercial applications and products.

## Abbreviations

AF: as fed
BCS: body condition score
BW: body weight
FW: free water
ICP-OES: inductively coupled plasma optical emission spectroscopy
MW: Metabolic water
NW: nutrient-enriched water
S_osmo_: serum osmolality
TW: tap water
TLI: total liquid intake
U_osmo_: urine osmolality
U_SG_: urine specific gravity.
